# Ciliopathy Is Differentially Distributed in the Brain of a Bardet-Biedl Syndrome Mouse Model

**DOI:** 10.1371/journal.pone.0093484

**Published:** 2014-04-02

**Authors:** Khristofor Agassandian, Milan Patel, Marianna Agassandian, Karina E. Steren, Kamal Rahmouni, Val C. Sheffield, J. Patrick Card

**Affiliations:** 1 Department of Neuroscience, the University of Pittsburgh, Pittsburgh, Pennsylvania, United States of America; 2 Department of Medicine, the University of Pittsburgh, Pittsburgh, Pennsylvania, United States of America; 3 Departments of Pharmacology and Internal Medicine, the University of Iowa, Iowa City, Iowa, United States of America; 4 Department of Pediatrics, the University of Iowa, Iowa City, Iowa, United States of America; 5 Howard Hughes Medical Institute, University of Iowa, Iowa City, Iowa, United States of America; The Ohio State University, United States of America

## Abstract

Bardet-Biedl syndrome (BBS) is a genetically heterogeneous inherited human disorder displaying a pleotropic phenotype. Many of the symptoms characterized in the human disease have been reproduced in animal models carrying deletions or knock-in mutations of genes causal for the disorder. Thinning of the cerebral cortex, enlargement of the lateral and third ventricles, and structural changes in cilia are among the pathologies documented in these animal models. Ciliopathy is of particular interest in light of recent studies that have implicated primary neuronal cilia (PNC) in neuronal signal transduction. In the present investigation, we tested the hypothesis that areas of the brain responsible for learning and memory formation would differentially exhibit PNC abnormalities in animals carrying a deletion of the Bbs4 gene (Bbs4^-/-^). Immunohistochemical localization of adenylyl cyclase-III (ACIII), a marker restricted to PNC, revealed dramatic alterations in PNC morphology and a statistically significant reduction in number of immunopositive cilia in the hippocampus and amygdala of Bbs4^-/-^ mice compared to wild type (WT) littermates. Western blot analysis confirmed the decrease of ACIII levels in the hippocampus and amygdala of Bbs4^-/-^ mice, and electron microscopy demonstrated pathological alterations of PNC in the hippocampus and amygdala. Importantly, no neuronal loss was found within the subregions of amygdala and hippocampus sampled in Bbs4^-/-^ mice and there were no statistically significant alterations of ACIII immunopositive cilia in other areas of the brain not known to contribute to the BBS phenotype. Considered with data documenting a role of cilia in signal transduction these findings support the conclusion that alterations in cilia structure or neurochemical phenotypes may contribute to the cognitive deficits observed in the Bbs4^-/-^ mouse mode.

## Introduction

BBS is a pleiotropic autosomal recessive disorder first described almost 150 years ago on the basis of excess adiposity, genital dystrophy, retinitis pigmentosa, mental deficiency, renal abnormalities, polydactyly and learning disabilities [1]–[3]. Contemporary research has established BBS as a complex phenotype derived from mutations in any of seventeen genes [4]–[7]. Protein products of seven of these genes (BBS1, BBS2, BBS4, BBS5, BBS7, BBS8) and another protein (BBIP10) form a complex known as the BBSome [8]–[10] that has been implicated in trafficking of membrane proteins to and from the cilia [11], [12]. The G protein-coupled somatostatin receptor 3 (SST3) [11] is among proteins transported to primary cilia by the BBSome while the dopamine receptor 1 requires BBSome proteins for translocation of the receptor from cilia [12]. Recent investigations have demonstrated that transport of melanin-concentrating hormone receptor 1 (MCHR1) and SST3 to cilia is compromised in Bbs2^-/-^ and Bbs4^-/-^ mice [13] and that impaired BBSome assembly contributes to the etiology of BBS phenotypes associated with the loss of function of BBS6, BBS10 and BBS12 genes [14]. Collectively, these data a) are consistent with a signal transduction function for cilia, b) support the conclusion that the BBSome enables such communication through its integral role in protein transport between cilia and parent neurons, and c) raises the possibility that interference with BBSome-mediated transport may contribute to the BBS disease phenotype.

A considerable body of literature supports the conclusion that deletions or mutations of BBS genes in the mouse genome produce animals with symptoms similar to the human disease phenotype. Early studies demonstrated that knock-out mice lacking either the Bbs2 or Bbs4 genes express many of the major symptoms of BBS [15]–[19]. Although there are variations in the expression of secondary symptoms of the human disorder in these Bbs^-/-^ animals (*e.g.,* hypertension; [19]), the core symptoms of the disease largely mimic those observed in the human disease. Importantly, behavioral alterations consistent with the cognitive impairments characteristic of the human disease have been documented in Bbs^-/-^ mice (e.g., reduced social dominance [17], [20], and elevated anxiety [20]), which is consistent with the reported increase in anxiety and depression in BBS children [21].

Surprisingly little is known about the neuropathological changes that are causal for the cognitive impairments that characterize BBS. Volume loss in the neocortex and hippocampus has been documented in a quantitative magnetic resonance imaging (MRI) study of the brains of 10 BBS patients [22], an observation that has also been validated in a Bbs1 knock-in mouse model by Davis and colleagues [23]. Davis et al also documented defective motile cilia on the ependymal lining of the third ventricle using transmission electron microscopy, and defects in cilia of the choroid plexus, subfornical organ and ventricular ependyma were subsequently documented in Bbs1 knock-in mutant mice by Swiderski et al [6]. In addition, Carter and colleagues identified signaling defects in a specific class of subventricular zone neural progenitor cells in Bbs1 mutant mice [24] and it was recently reported that ectopic BBS4 rescues the BBS phenotype in Bbs4^-/-^ mice [25]. Nevertheless, the morphology and distribution of PNC has not been comprehensively investigated in Bbs animal models.

In the present study we tested the hypothesis that mice carrying a deletion in the *Bbs4* gene will exhibit pathological changes in the number and morphology of primary neuronal cilia in areas of the brain involved in learning and memory, but not in areas that do not contribute to this phenotype. This hypothesis is based upon the accumulating evidence that PNC are ubiquitously distributed throughout the brain [26], are involved in neuronal signal transduction [27], that the BBSome complex sorts membrane proteins to cilia [9], and that conditional ablation of cilia delays spatial learning through elimination of adult hippocampal stem/progenitor cells [28].

## Results

### 1.1. Conformation of neuropathology in the Bbs4^-/-^ mouse

Comparison of the general morphology of the brains of WT and Bbs4^-/-^ mice confirmed the gross differences in central nervous system structure (CNS) documented previously for the Bbs1 knock-in model [23] and Bbs deletion mutants [6]. Consistent with the findings of Swiderski and colleagues [6] we observed that deletion of the Bbs4 gene produced enlarged ventricles and reduced mass of the hippocampus and striatum compared to WT littermates.

### 1.2. Immunohistochemical localization of ACIII

To further confirm the specificity of the anti-ACIII antibody used for immunocytochemical localization of PNC we compared staining of primary cilia associated with neurons in the ventromedial nucleus of hypothalamus (VMH) to motile cilia on the luminal surface of the adjacent ependymal lining of the third ventricle. Immunohistochemical localization of ACIII revealed immunopositive PNC in the VMH and in all regions of the neuraxis in WT mice. However, no immunopositive staining of cilia was observed on the luminal surface of the ependyma (data not shown). We also conducted preabsorbtion controls that confirmed the observations of Bishop and colleagues using the same antiserum [26].

Qualitative analysis of wild type and Bbs4^-/-^ brains revealed apparent differences in the number and morphology of ACIII immunopositive PNC. These differences were particularly prominent in regions of the brain involved in cognition and memory function of all Bbs4^-/-^ animals. Substantial reductions in both the number and morphology of immunopositive PNC in Bbs4^-/-^ mice were observed within amygdala and hippocampus. However, careful examination of subcortical regions revealed that the ciliopathy was not a uniform property of all CNS cell groups. On the basis of these initial qualitative observations we designed a focused quantitative analysis to determine the extent and reproducibility of the ciliopathy. This analysis was conducted on the amygdala, hippocampus, and four subcortical regions (nucleus accumbens shell, suprachiasmatic nucleus, principal sensory trigeminal nucleus and the dorsal tegmental nucleus of Gudden). The analysis of each of these regions was compared in Bbs4^-/-^ mice and wild type littermates and the areas of analysis were standardized across animals.

Quantitative comparisons revealed that the number of immunopositive cilia (cilia/μm^2^) was significantly reduced in the brain of Bbs4^-/-^ mice. Statistical analysis of all brain areas combined using repeated measures ANOVA demonstrated a significant main effect of region with respect to both the number of cilia per unit area [F(19, 95)  = 41.44, P<0.001] as well as cilia length [F (11,55)  = 9.11, P<0.001]. The ANOVA also revealed a significant interaction effect between brain region and genotype, for cilia number [F (19, 95 = 11.47, P<0.001] and cilia length [F (11,55) = 5.24, P<0.001]. The data that these observations were drawn from are described below.

#### 1.2.1. Cilia of amygdala

**Figure 1:**
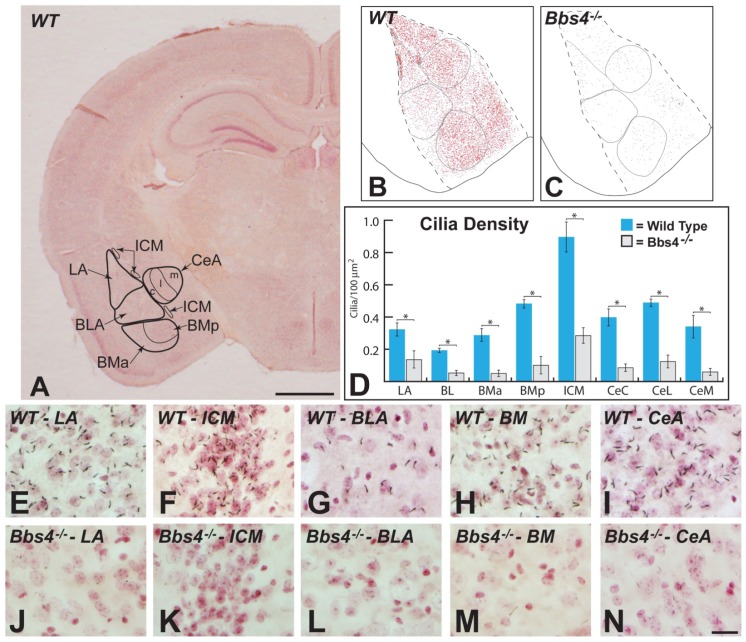
The areas of the amygdala sampled and the distributions of ACIII immunopositive cilia in subdivisions of amygdala in WT and Bbs4^-/-^ mice are illustrated. Figure A shows the subdivisions of amygdala and the coronal plane that was analyzed in this study. Figures B and C are maps documenting distribution of cilia within these regions of WT (B) and Bbs4^-/-^ (C) brains that were acquired at high magnification using Sterioinvestigator image analysis software. Figure D shows the density of cilia ± the SEM within each amygdala subdivision of WT (blue) and Bbs4^-/-^ (gray) animals; asterisks and brackets indicate where statistical analysis demonstrated statistical differences between WT and Bbs4^-/-^ mice. The data for (D) was collected from three WT and five Bbs4^-/-^ mice. High magnification comparisons of the distribution of cilia within comparable regions of each of the amygdala subdivisions are demonstrated in figures E through N. Note the association of cilia with neurons counterstained with neutral red in each region as well as the dramatic reduction in ACIII+ cilia in Bbs4^-/-^ animals. BLA  =  basolateral nucleus of amygdala; BMa  =  basomedial amygdala, anterior part; BMp  =  basomedial amygdala, posterior part; CeA  =  central nucleus of amygdala with capsular (c), lateral (l) and medial (m) subdivisions; ICM  =  intercalated cell masses; LA  =  lateral nucleus of amygdala. Scale bar in figure A  =  1 mm, in N  =  20 μm; figures E – N are of the same magnification.

ACIII immunoreactivity (IR) was observed in PNC throughout all the subdivisions of amygdala in WT animals (Figures 1 A, B, E–I). Immunopositive profiles were homogeneously distributed, dense, and IR appeared to extend throughout the full length of each cilium. In addition, ACIII+ PNC were associated with neurons in each amygdala subdivision. Analysis of the number of IR cilia per unit area in WT mice revealed statistically significant differences among amygdala subdivisions, particularly in relation to the high density of IR cilia observed in the intercalated cell masses (ICM) (Figure 1D). As detailed in the Methods section we normalized observations between WT and mutant animals by dividing the number of immunopositive cilia counted by the area (cilia/μm^2^) and reporting the distribution as ciliary density. Quantitative analysis revealed an average density measure of 0.90 cilia/100 μm^2^ ±0.10 in the ICM, a number that far exceeded that documented in all other amygdala subdivisions and was statistically significant (Figure 1D; P<0.000005). This difference is likely related to the high packing density of ICM neurons relative to other amygdala subfields. Statistical analysis also revealed significant differences between other amygdala subdivisions of WT animals. After the ICM, the highest cilia number was observed in the posterior portion of the basomedial nucleus with a number that was significantly higher than that observed in the lateral, basolateral and anterior basomedial nucleus. The number of ACIII+ cilia per unit area in the lateral and capsular subdivisions of the central nucleus of the amygdala also was significantly higher than that observed in lateral, basolateral and anterior subdivision of the basomedial nucleus (Figure 1 D).

Strikingly, immunopositive cilia in each amygdala subdivision of Bbs4^-/-^ mice were dramatically reduced compared to WT controls (compare figures 1 B with 1 C and 1 E–I with 1 J–N). The cardinal features that separated Bbs4^-/-^ and WT groups were the dramatic reduction in immunopositive profiles in Bbs4^-/-^ animals and reduced intensity of immunocytochemical signal. These differences were striking and consistent in all Bbs4^-/-^ mice. In this regard, it is important to note that the staining of immunopositive cilia in Bbs4^-/-^ animals was generally much lighter than that observed within WT littermates, even though processing procedures for tissue sections from both groups were standardized. Statistical analysis revealed that the reductions in immunopositive profiles were highly significant in all subdivisions of the amygdala in Bbs4^-/-^ animals compared to WT littermates (Figure 1D). Furthermore, the reductions were consistent across amygdala subdivisions; *e.g.*, no subdivision of the amygdala was spared the reduction of immunopositive profiles. To determine if the reduction in immunopositive cilia was a consequence of reductions in the number of neurons we conducted an unbiased stereological sampling. This analysis revealed no statistical difference between Bbs4^-/-^ mice and their WT littermates. Thus, the reduction in the number of immunopositive cilia within sampled amygdala subdivisions cannot be attributed to a reduced number of neurons. However, it is important to emphasize that this analysis was conducted on only a single section through the amygdala (Figure 1A) and did not sample the rostrocaudal extent of each subfield. Accordingly, we cannot exclude the possibility that neuronal loss occurred within the amygdala.

**Figure 2:**
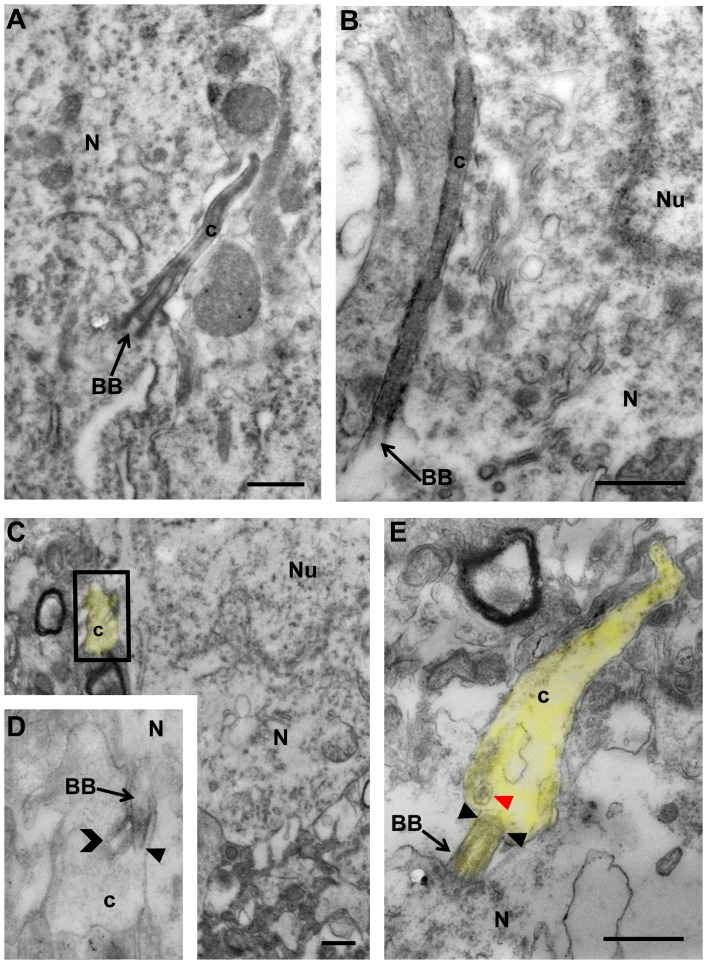
Transmission electron microscopic images of PNC in amygdala of WT (A & B) and Bbs4^-/-^ mice (C & E). Note the intact basal bodies associated with neurons in WT and Bbs4^-/-^ mice and abnormal cilia starting from basal bodies in C & E compared to healthy PNC with microtubules in A & B. The insert (D) is a higher magnification image of the area incorporated in the rectangle on (C). The yellow highlights the shape of PNC in Bbs4^-/-^ lateral amygdala. The solid arrowheads in D and E point to the ciliary transition zones; open head arrow points to the broken axoneme; red arrow head points to the spherical inclusion bodies. The yellow highlights the shape of PNC in Bbs4^-/-^ lateral amygdala. The data was collected from three WT and five Bbs4^-/-^ mice. BB – basal bodies; c – cilium; N -neuron; Nu – nucleus; arrows point to BB. Scale bars: 0.5 μm for A-E.

Electron microscopic analysis documented dramatic alterations in the ultrastructure of PNC in Bbs4^-/-^ mice compared to WT littermates. Cilia of WT mice contained 9 pairs of microtubules arising from the basal body and extending throughout the full extent of each organelle (Figures 2 A & B). In contrast, PNC of Bbs4^-/-^ mice exhibited gross pathological changes. Whereas the basal bodies of Bbs4^-/-^ PNC appeared normal, marked abnormalities were apparent beyond the transition zone (Figures 2 D & E). In the regions immediately distal to basal bodies radial spokes were often broken. Beyond the transition zone the cilia were truncated and exhibited highly disorganized microtubules that could only be recognized by virtue of occasional continuity with basal bodies (Figures 2 C & E). However, the most distal portions of the cilia exhibited the most prominent pathological changes, characterized by pronounced swelling almost entirely lacking in microtubules and the presence of spherical inclusion bodies (Figure 2E).

#### 1.2.2. Cilia of hippocampi

**Figure 3:**
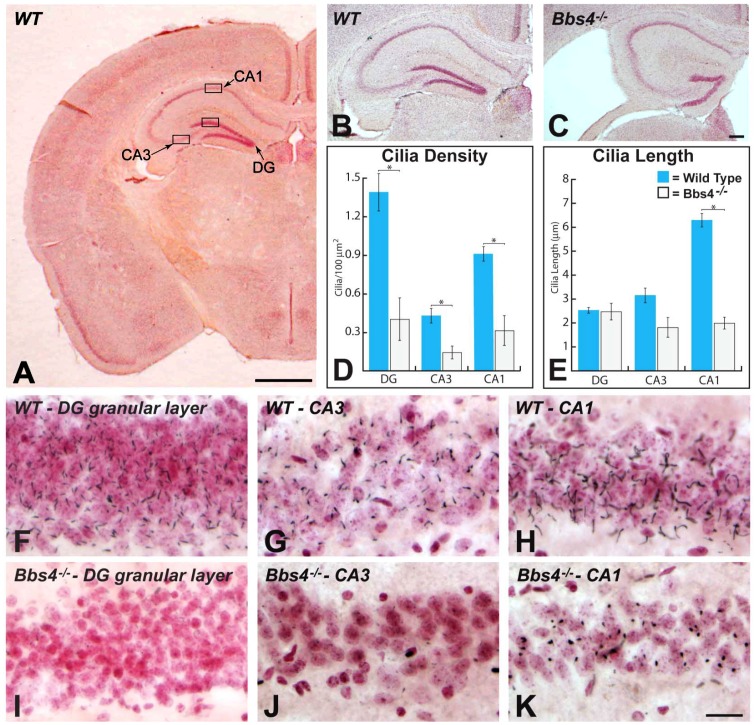
The areas of rostral (dorsal) hippocampus sampled and the distributions of ACIII immunopositive PNC in subdivisions of the hippocampus in WT and Bbs4^-/-^ mice are illustrated. Figure A shows the subdivisions of hippocampus in coronal plane of chosen in this study for sampling. The boxed areas define the regions in which quantitative data was collected for each area. Figures B and C illustrate the cytoarchitecture of WT (B) and Bbs4^-/-^ (C) hippocampus at this plane of section. Note the reduction in hippocampal mass and the resulting expansion of the lateral ventricle in Bbs4^-/-^ mouse brain. Figures D and E illustrates the density of cilia ± the SEM (D) and length of cilia ± the SEM within each of the analyzed hippocampal subfields of WT (blue) and Bbs4^-/-^ (gray) mice; asterisks and brackets indicate where statistical analysis demonstrated statistical differences between WT and Bbs4^-/-^ mice. The data for (D) and (E) was collected from three WT and five Bbs4^-/-^ mice. High magnification comparisons of the distribution of cilia within these regions of hippocampus are shown in figures F through K. Note the association of cilia with neurons counterstained with neutral red in each region as well as the dramatic reduction in ACIII+ cilia in Bbs4^-/-^ animals. *Cornu Ammonis* areas: CA1, CA3 and DG (dentate gyrus). Scale bar in figure A = 1 mm, in K = 20 μm and figures F – K are of the same magnification.

**Figure 4:**
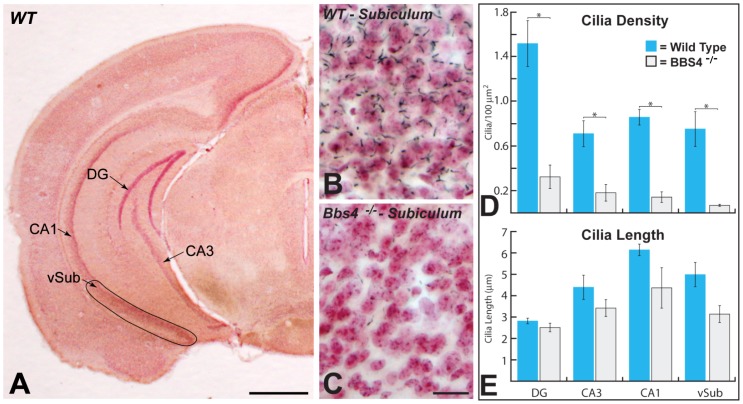
The areas of caudal (ventral) hippocampus sampled and the distributions of ACIII immunopositive PNC in hippocampal subdivisions of WT and Bbs4^-/-^ mice are illustrated. Figure A illustrates the subdivisions of hippocampus sampled at this caudal coronal plane. The arrows indicate the regions in which quantitative data was collected for each area. Figures B and C illustrate the distribution of cilia within ventral subiculum of WT (B) and Bbs4^-/-^ (C) mice in this plane of section. Note the association of cilia with neurons counterstained with neutral red in each region as well as the dramatic reduction in ACIII+ cilia in Bbs4^-/-^ animals. Figures D and E illustrates the density of cilia ± the SEM (D) and length of cilia ± the SEM (E) within each of the analyzed hippocampal subfields of WT (blue) and Bbs4^-/-^ (gray) mice; asterisks and brackets indicate where statistical analysis demonstrated statistical differences between WT and Bbs4^-/-^ mice. The data for (D) and (E) was collected from three WT and five Bbs4^-/-^ mice. *Cornu Ammonis* areas: CA1, CA3 and DG (dentate gyrus); vSub  =  ventral subiculum. Scale bar in figure A = 1 mm, in C = 20 μm and figures B & C are of the same magnification.

ACIII immunopositive profiles were prevalent throughout all major subdivisions of hippocampal formation (DG, CA3, CA1, and subiculum) of WT animals (Figures 3 and 4). Among subdivisions, immunopositive PNC were most prevalent within the granule cell layer of dentate gyrus (Figure 3F), stratum pyramidale of CA3 & CA1 (Figures 3 G & H), and the deep cell layer of the subiculum (Figure 4B) that expands from the termination of CA1 stratum pyramidale. The dense concentration of immunopositive cilia within these cell layers contrasted with the sparse distribution of immunoreactive profiles in stratum oriens, stratum radiatum and stratum lacunosum moleculare of CA3 & CA1 and the cell sparse external portion of the subiculum. Nevertheless, it is important to note that cilia were associated with interneurons known to populate those regions.

We conducted quantitative analyses of the density of PNC within standardized subregions of the dentate granule cell layer and stratum pyramidale of CA3 and CA1 in sections through rostral (dorsal; Figure 3A) and caudal (ventral; Figure 4A) hippocampi, and in the deep pyramidal cell layer of ventral subiculum (see designated areas in figures). The density of PNC per unit area within these regions in WT animals was highest within the dentate granule cell layer in both the rostral and caudal hippocampus, which is likely reflective of the smaller size and high packing density of neurons within this cell layer relative to the other cell groups. Comparison of PNC number per unit area in CA3 and CA1 of WT mice revealed a higher density measure in CA1, particularly at rostral levels of hippocampi (Figure 3H). However, with one exception (CA1 vs. ventral subiculum) statistical analysis revealed that significant differences were restricted to comparisons between the dentate granular cell layer relative to CA3 (rostral and caudal hippocampus) and ventral subiculum.

Dramatic reductions in the number of ACIII immunopositive profiles were observed in all subdivisions of the hippocampus of Bbs4^-/-^ animals relative to WT littermates (compare Figures 3 I–K and 4 C to 3 F–H & 4 B). Quantitative statistical analysis confirmed that these reductions were highly significant and involved all hippocampal subdivisions (Figure 3 D and 4D).

The morphology of immunopositive profiles in the cell dense layers of dorsal and ventral hippocampi of knockout animals displayed prominent structural differences compared to WT animals. This was particularly apparent in the CA1 region of rostral (dorsal) hippocampi where PNC in the Bbs4^-/-^ mice characteristically appeared truncated relative to those in comparable regions of WT mice (compare figures 3 K & H). We therefore conducted a quantitative analysis of the length of immunopositive cilia within each region. Interestingly, this analysis revealed a significant difference in the length of ACIII immunopositive cilia in the stratum pyramidale of CA1 in the rostral (dorsal) hippocampus compared to the caudal (ventral) hippocampus of Bbs4^-/-^ mice and also documented a difference in length of ACIII immunopositive cilia in the dorsal and ventral hippocampus of WT animals (Figures 3 E and 4 E). In WT mice immunopositive cilia in CA3 were longer in the ventral hippocampus compared to the dorsal hippocampus. However, there was no significant reduction in the length of immunopositive profiles in either area of Bbs4^-/-^ mice. Similarly, there was no significant difference in the length of immunopositive profiles in CA1 of WT mice in the dorsal and ventral hippocampus (6.29±0.30 μm in dorsal and 6.15±0.24 μm in ventral). In contrast, a statistically significant reduction in cilia length was observed in CA1 of the dorsal hippocampus of Bbs4^-/-^ animals compared to WT control animals (P = 0.00023; Figure 3E).

**Figure 5:**
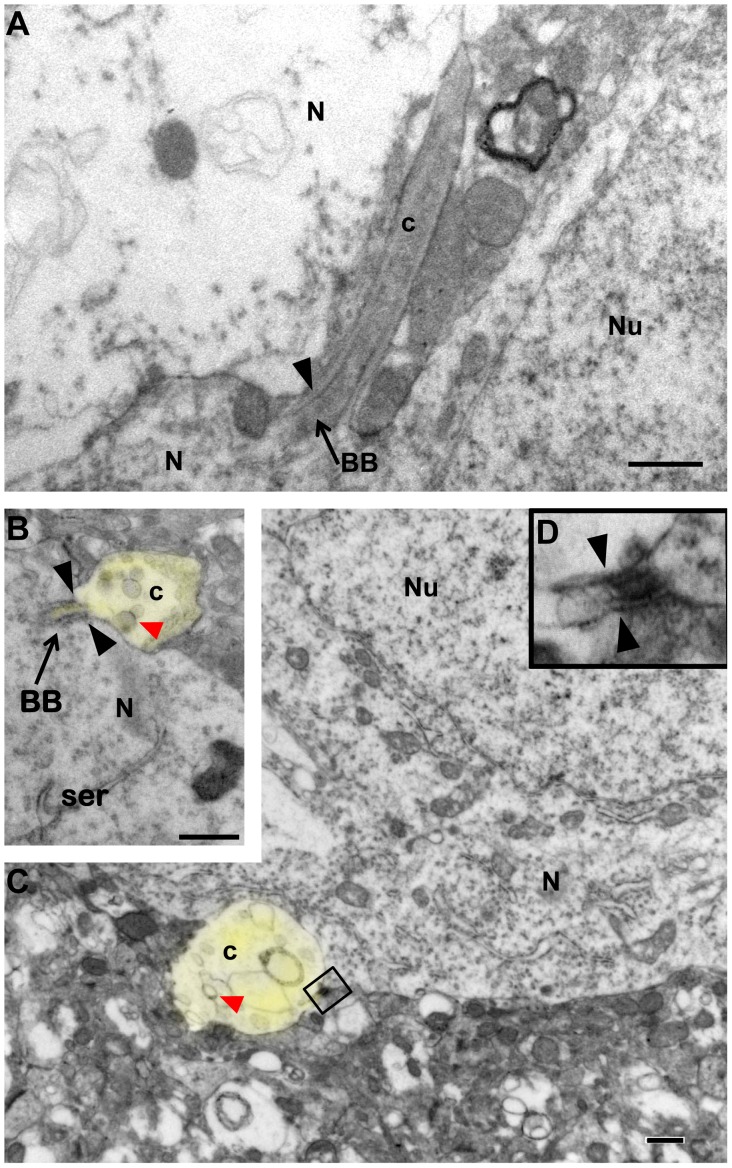
Transmission electron microscopic images of PNC in CA1 hippocampus of WT (A) and Bbs4^-/-^ mice (B & C). Note the intact basal bodies associated with neurons in WT and Bbs4^-/-^ mice and abnormal balloon-like cilia starting from basal bodies in B & C compared to healthy PNC with microtubules in A. The insert (D) is a higher magnification image of the area incorporated in the rectangle on (C). The yellow highlights the shape of PNC in Bbs4^-/-^ hippocampus. The data was collected from three WT and five Bbs4^-/-^ mice. BB – basal bodies; c – cilium; N - neuron; Nu – nucleus; arrows point to BB; solid arrow heads point to the ciliary transition zones; red arrow heads point to the spherical inclusion bodies. Scale bars: 0.5 μm for A–C.

As in the amygdala, electron microscopic analysis revealed marked alterations in cilia ultrastructure of Bbs4^-/-^ compared to littermate controls (Figures 5 B & C). This analysis focused upon CA1 at rostral levels of hippocampus. Few alterations were observed in the structure of basal bodies but the regions beyond the transition zone revealed marked structural alterations that recapitulated those observed in the amygdala (Figures 5 B & D). Particularly characteristic of these changes were truncated length, swollen balloon-like morphology and a paucity of microtubules. Unusual spherical inclusion bodies were also prevalent in these pathological profiles (Figures 5 B & C).

In each of the regions subjected to quantitative analysis of the number of immunopositive cilia we also analyzed the numbers of neurons. Within these restricted regions, which were subsets of the hippocampal subfields, unbiased stereological analysis revealed no statistical difference in neuron number. However, once again, it is important to emphasize that our analysis was confined to restricted regions of each hippocampal subfield within a single section. Examination of the neutral red counterstained sections revealed a clear loss of hippocampal mass at rostral levels and we cannot preclude the possibility that there was also neuron loss in caudal hippocampi.

#### 1.2.3. Subcortical regions of Bbs4^-/-^ brains

**Figure 6:**
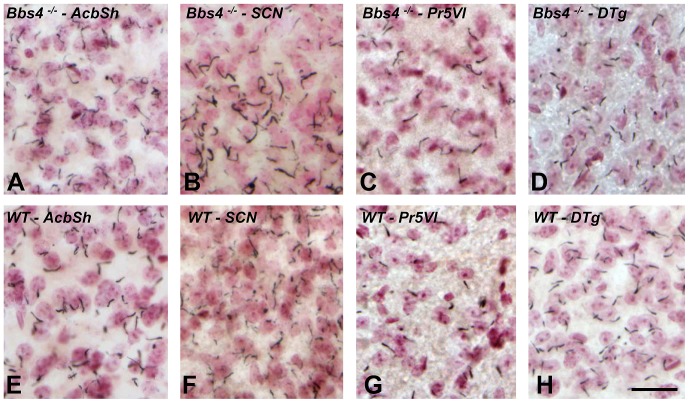
Similar distribution of ACIII immunopositive PNC in Bbs4^-/-^ (A–D) compared to WT (E–H) regions of the brain is illustrated. AcbSh – nucleus accumbence shell; SCN – suprachiasmatic nucleus; Pr5Vl - principal sensory trigeminal nucleus, ventrolateral subdivision; DTg - dorsal tegmental nucleus. The data was collected from three WT and five Bbs4^-/-^ mice. Scale bars =  25 μm for A*-H.*

**Figure 7:**
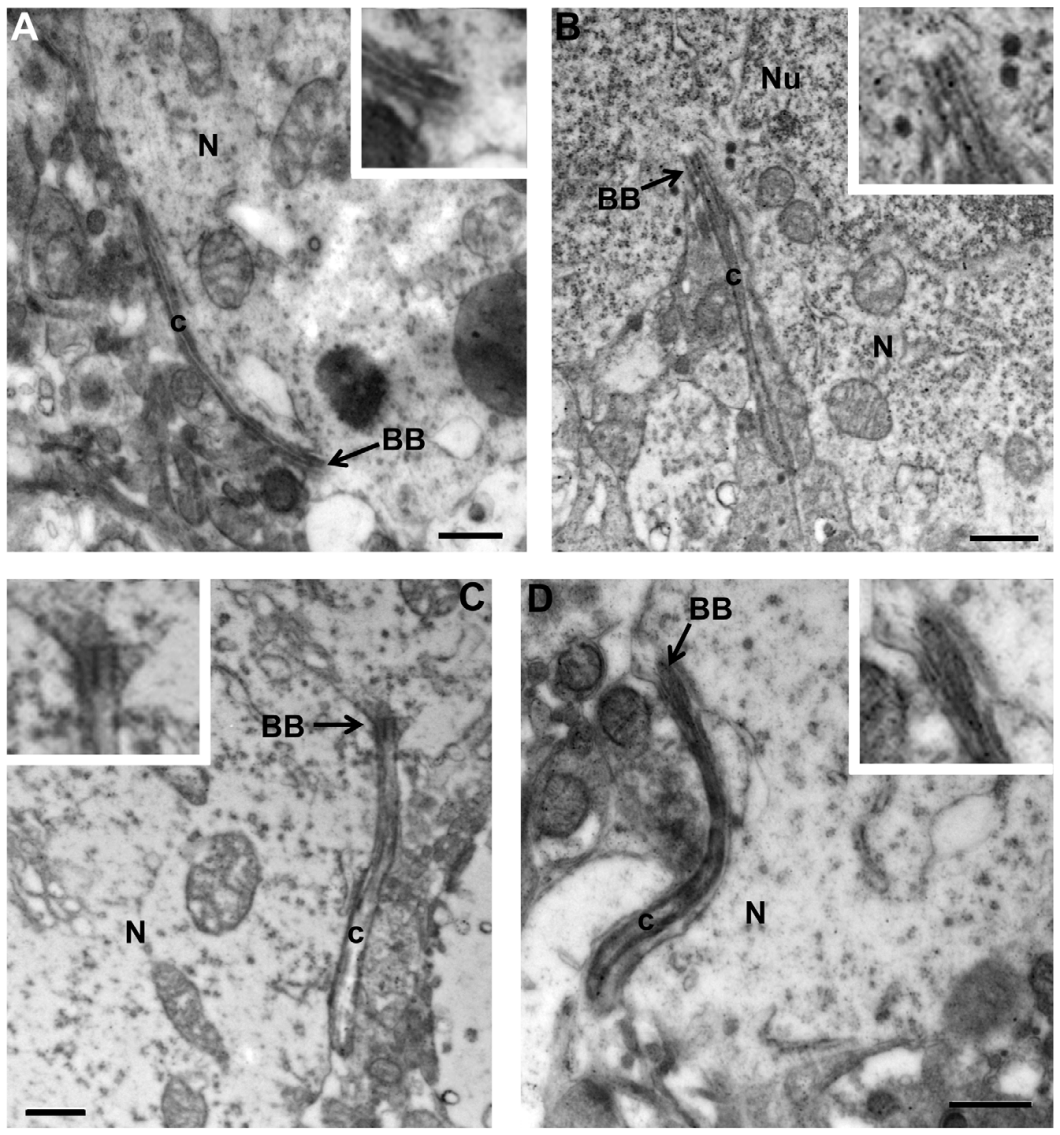
Transmission electron microscopic images of PNC in AcbSh (A), SCN (B), Pr5Vl (C) and DTg (D) of Bbs4^-/-^ mice. The morphologically intact elongated PNC containing axonemal microtubules and their basal bodies associated with neurons in four areas not known to contribute to the human phenotype or functional deficits in the Bbs4^-/-^ animal model are illustrated. Insets in each figure demonstrate the basal bodies of the cilia at higher magnification. No overt differences in morphology of PNC in these regions were observed compared to those of wild type animals (see Figure 2). The data for was collected from three WT and five Bbs4^-/-^ mice. BB – basal bodies; c – cilium; N -neuron; Nu – nucleus; arrows point to BB. Scale bars: 0.5 μm for A-D. Abbreviations as for Figure 6.

**Figure 8:**
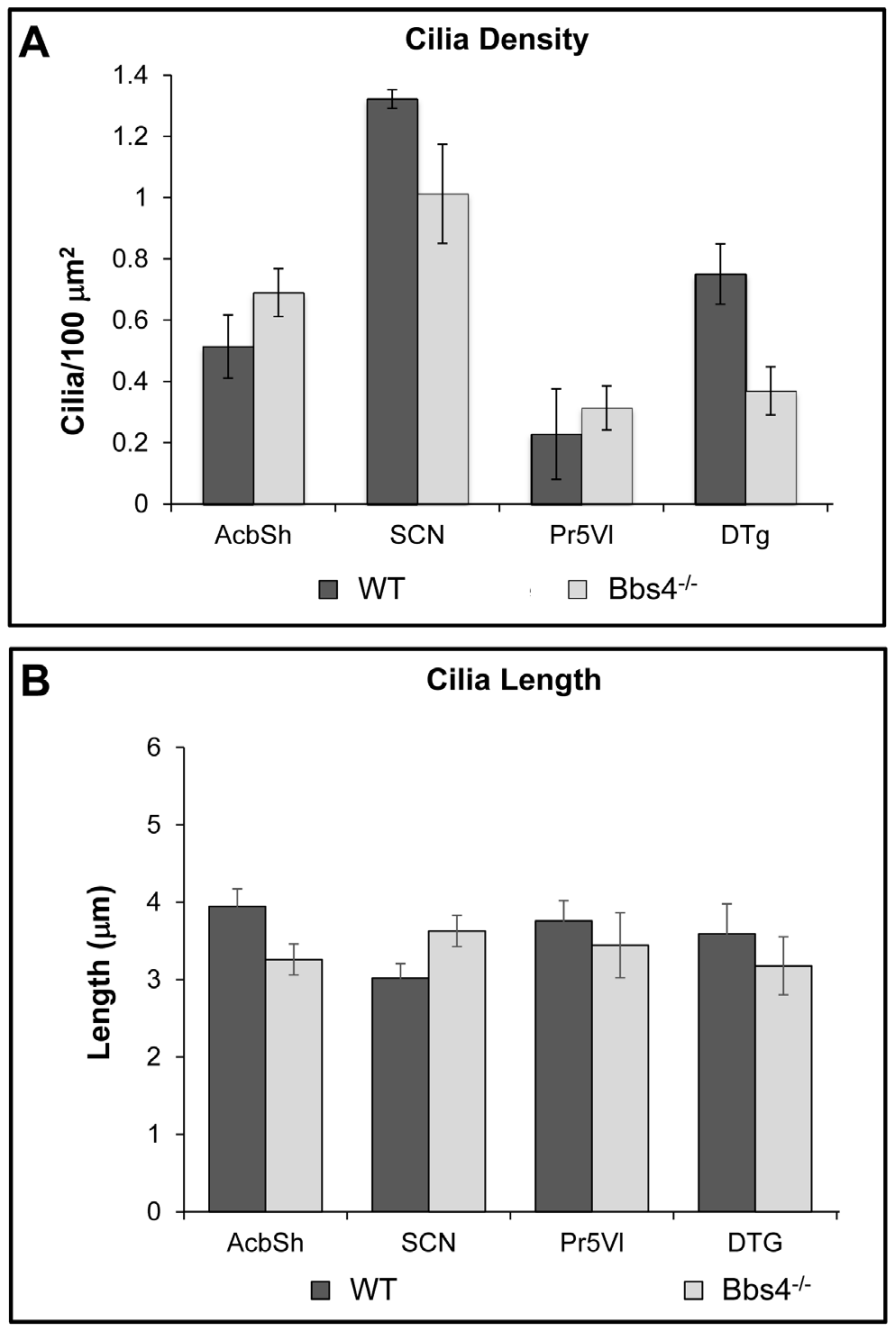
Statistical analysis of brain regions not affected by the syndrome compared to the corresponding areas in WT mice: cilia length ± the SEM (A) and cilia density ± the SEM (B) within each of the analyzed brain regions of WT (dark gray) and Bbs4^-/-^ (light gray) mice. Abbreviations as for Figure 6. The data for (A) and (B) was collected from three WT and five Bbs4^-/-^ mice.

**Figure 9:**
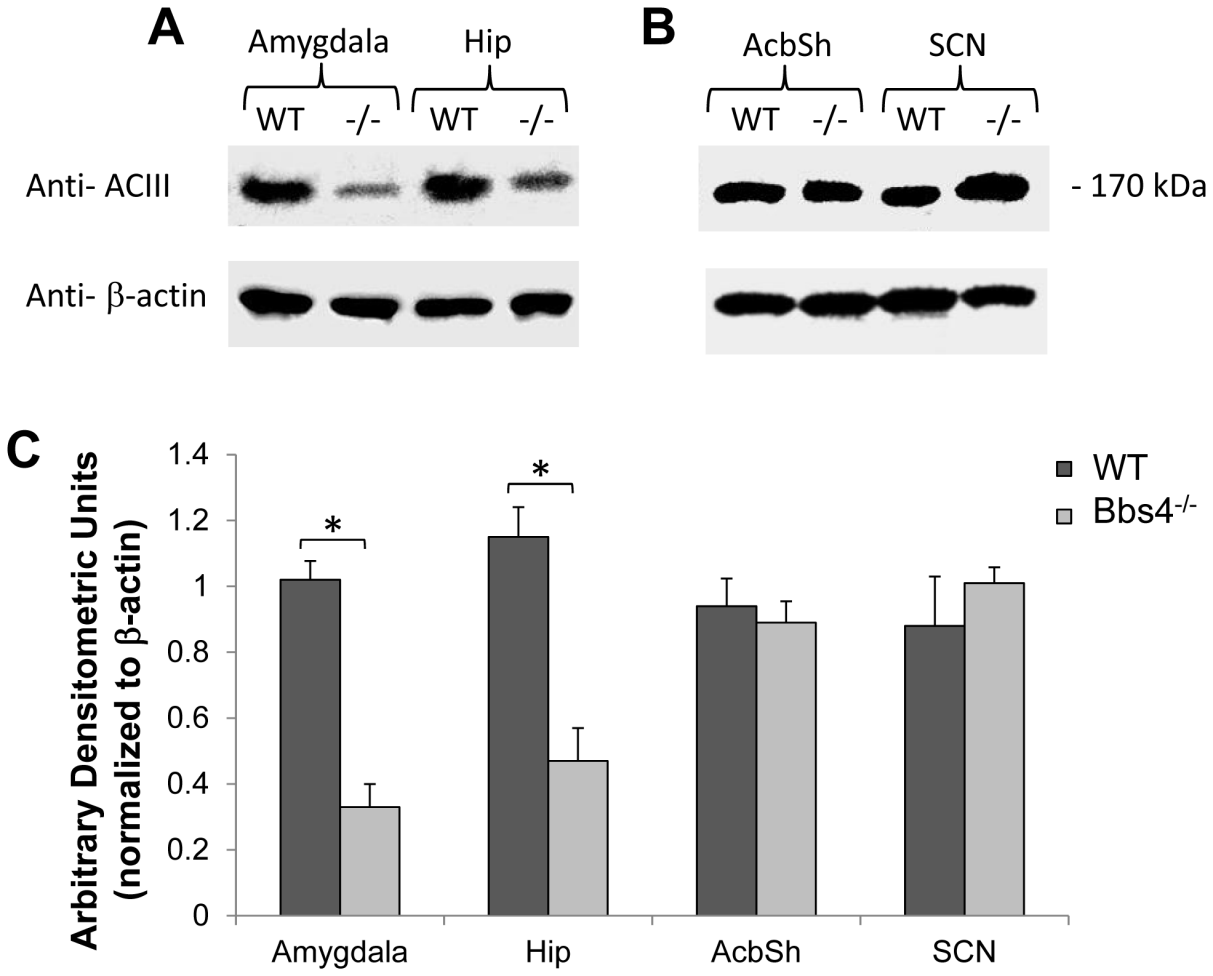
Western blot analysis of ACIII expression in hippocampus, amygdala, AcbSh and SCN of WT and Bbs4^-/-^ mice brains normalized for actin. Note the lower level of ACIII in hippocampus and amygdala of Bbs4^-/-^ mice compared to WT (A) and no changes in ACIII expression in AcbSh and SCN (B). Shown is a representative western blot from three separate experiments. The data for western blot was collected from three WT and three Bbs4^-/-^ mice. Densitometric analysis (C) ± the SEM of the immunoblots shows the amounts of immunoreactive ACIII in the dissected areas of WT mice brains (dark gray) *versus* the corresponding dissected areas of Bbs4^-/-^ mice brains (light gray) using densitometric values after correction for the levels of b-actin (*p<0.05). The data for Western blot was collected from three WT and three Bbs4^-/-^ mice.

**Figure 10:**
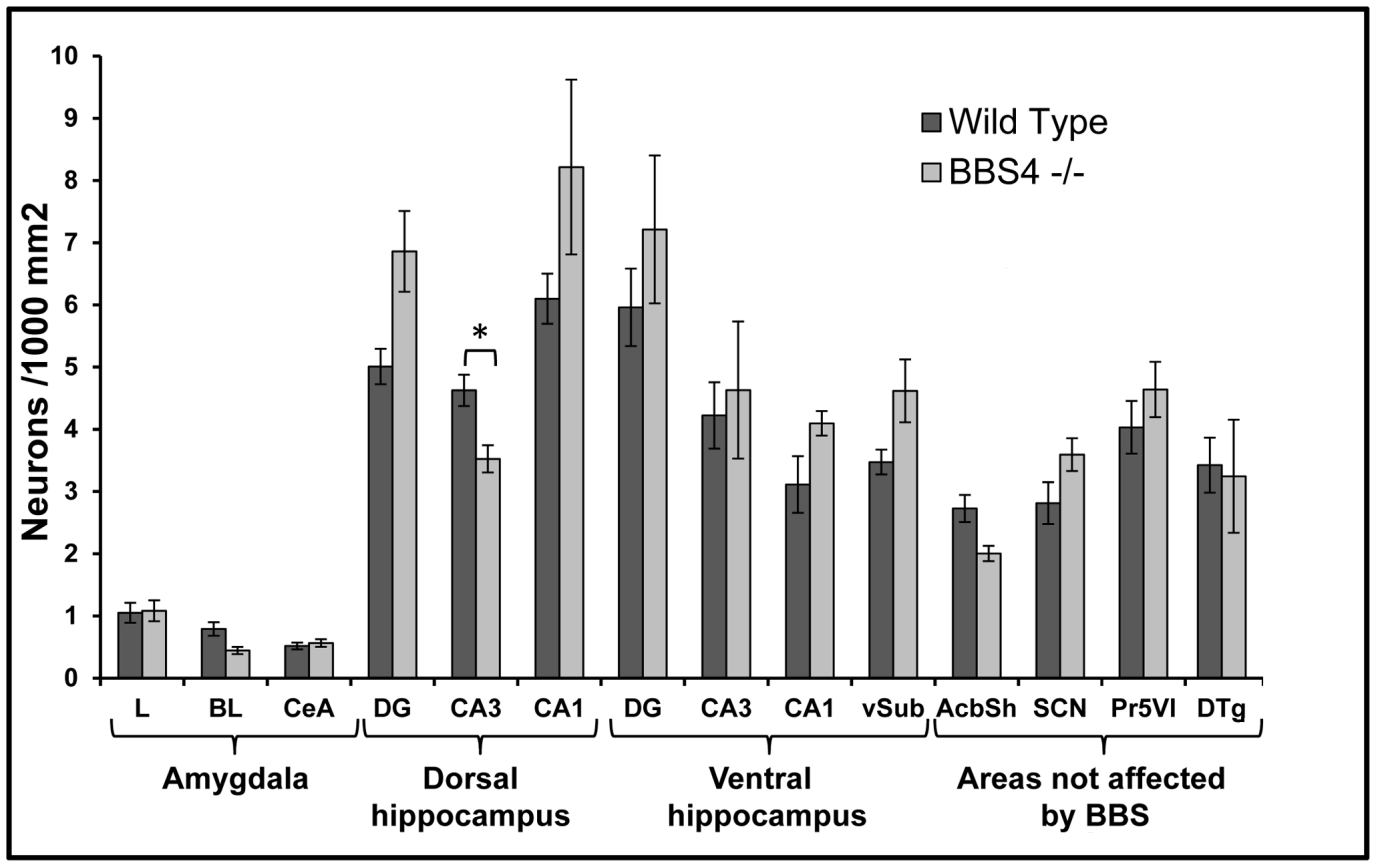
Neuronal density ± the SEM within each of the analyzed subfields of amygdala, hippocampus and regions not affected by the syndrome in WT (dark gray) and Bbs4^-/-^ (light gray) mice brains. Note the absence of a significant difference in the number of neurons in all described regions except for the CA3 of dorsal hippocampus. The data was collected from three WT and four Bbs4^-/-^ mice. Abbreviations as for Figures 1, 4 & 6.

Careful analysis revealed that ciliopathy was not a uniform property of the Bbs4^-/-^ mouse brain. Qualitative light microscopic analysis of regions of the brain that are not linked to the BBS phenotype in Bbs4^-/-^ mutant mice showed no apparent alterations in either the number or morphology of primary neuronal cilia. It is important to note that unpublished data collected by one of us (Rahmouni) has demonstrated *Bbs4* gene expression in all of the areas that were subject to analysis in this study. Subcortical areas with no known association with the BBS phenotype included regions mediating behavioral state (*e.g.*, suprachiasmatic nucleus), sensation (*e.g.*, principal sensory trigeminal nucleus), olfaction (*e.g.*, olfactory tubercle), audition (*e.g.*, ventral cochlear nucleus and inferior colliculus), vestibular function and orientation (dorsal tegmental nucleus), motor function (*e.g.*, lateral globus pallidus), reinforcement (*e.g.*, nucleus accumbens), as well as multimodal processors important for the maintenance of homeostasis and responsiveness to stress (*e.g.*, bed nucleus of stria terminalis). Diffusely projecting, multifunctional areas of the CNS (e.g., pedunculopontine nucleus) also showed no apparent ciliopathy. A subset of these regions was subject to quantitative analysis. They included the nucleus accumbens shell (AcbSh), suprachiasmatic nucleus (SCN), principal sensory trigeminal nucleus, ventrolateral subdivision (Pr5VL) and dorsal tegmental nucleus of Gudden (DTg). Our prediction that the areas not related to the BBS phenotype would not display PNC abnormalities was confirmed morphologically on light microscopic (Figure 6), electron microscopic levels (Figure 7), statistically (Figure 8) and biochemically (Figure 9). Similarly, unbiased stereological analysis of numbers of neurons within all regions of the neuraxis did not reveal statistically significant differences between WT and mutant mice except for the CA3 area of dorsal hippocampus (Figure 10).

### 1.3. Western blot analysis

Biochemical analysis, carried out by an investigator masked to the samples, revealed statistically significant reductions in levels of ACIII in amygdala and hippocampus of Bbs4^-/-^ mice compared to the same areas of WT animals (Figure 9 A, C). In contrast, no statistically significant changes in ACIII levels were observed in the AcbSh and SCN between Bbs4^-/-^ and WT mice (B, C).

## Discussion

Developmental delay and intellectual impairment are cardinal features of ciliopathy disorders, including BBS [29], [30]. The type and degree of impairment evident in BBS is variable among individuals [21], [31], [32] and may be related to variable involvement of the genes causal for the disorder. A quantitative MRI study of 10 BBS patients, 8 of which contained a mutation in the BBS1 gene, documented a total gray matter volume loss of 10% with regional abnormalities concentrated in the orbitofrontal cortex, temporal lobe and hippocampus [22]. Neuroanatomical studies of mice with a knock-in mutation of the *Bbs1* gene have also revealed a marked loss in cortical and hippocampal mass similar to that documented in the human disease [23]. BBS deletion mutants lacking the *Bbs2*, *Bbs4* and *Bbs6* genes have recently demonstrated ventriculomegaly with an associated loss of cortical and hippocampal mass [6]. The data reported in the current study add to literature documenting the pathological consequences of *Bbs4* gene^-/-^ (Bbs4^-/-^) deletion by demonstrating dramatic alterations in the structure, apparent distribution, and neurochemistry of PNC. A developing literature now suggests that PNC play an important role in the generation of symptoms in a variety of human ciliopathies (*e.g.*, nephronophthisis, BBS, Orofacial-digital type 1, Meckel-Gruber, Joubert and Von Hippel-Lindau syndromes) [22], [33], [34]. Additionally, Keryer et al recently reported that huntingtin-associated protein 1 regulates ciliogenesis through an interaction with pericentriolar material 1 protein, implicating ciliopathy as a factor that may contribute to the pathogenesis of Huntington disease [35]. Our data bring further clarity to the extent of ciliopathy in the Bbs4^-/-^ mouse model of Bardet-Biedl syndrome and implicate ciliopathy as a potential contributing factor to the disease phenotype. Whether the pathological changes in PNC observed in our study are causally related to the deletion of the *Bbs4* gene remains to be determined. Similarly, we cannot ascribe a causal relationship between the ciliopathy documented in our study and the loss of function observed in Bbs4^-/-^ mice. Nevertheless, a number of studies in the literature are consistent with this conclusion. Notable in this regard is a recent study by Chamling and colleagues reporting that expression of the human *BBS4* gene in Bbs4 null mice rescued the majority of BBS related consequences of *Bbs4* gene deletion [25]. A number of other studies examining the signaling capacity of cilia suggest that this restoration of function is linked to the presence of the ACIII enzyme. ACIII is part of cAMP-dependent, G protein-coupled signaling cascade used in cell communication [27], [36], [37] and is an essential component in the olfactory system that converts chemical stimuli into electrical signals [38]. The presence of this second messenger in PNC has been influential in revealing the full extent of the distribution of PNC in the CNS [25] as well as for documenting a potential role for primary cilia in CNS-mediated functions. For example, Wang and colleagues recently demonstrated that ACIII is required for novel learning and extinction of contextual memory [39]. Furthermore, it is now well established that BBSome proteins play a an integral role in the transport of the SST3 and MCHR1 receptors to and from cilia [13] and that receptor expression can influence the degree of ACIII localization within cilia [40]. In this regard, overexpression of the 5HT6 receptor essentially eliminated the localization of ACIII and SST3 to cilia. Importantly, it has also been demonstrated that transport of SST3 to cilia is compromised in Bbs2^-/-^ and Bbs4^-/-^ mutant mice [13].

Additional evidence supports the conclusion that the disruption of ACIII localization observed in our study may contribute to the neuropathological alterations evident in the brains of BBS deletion mutants. In this regard Guadianna and colleagues [40] reported that ACIII is required for normal development of the dendritic trees of cortical projection neurons in mice. Similarly, Kumamoto and colleagues demonstrated that conditional deletion of cilia from dentate granule neurons born in the adult brain produces severe defects in dendritic development and in the synaptic integration of these neurons into hippocampal circuitry [41]. Although not examined in our study, these data imply that the ciliopathy and reduced ACIII staining observed in our investigation may be indicative of defects in the dendritic architecture of affected neurons. Such alterations, should they exist, would certainly be reflective of altered circuit dynamics in afflicted areas, a finding that would be consistent with the cognitive deficits documented in BBS mutant animals.

The regions in which ACIII immunopositive cilia are enriched in the WT brain and reduced in Bbs4^-/-^ mice are integral to learning and memory. Within the hippocampus they include the granule cells of dentate gyrus, stratum pyramidale of CA3 and CA1, and the subiculum. These neurons are essential for acquisition and consolidation of declarative memory and lesions of components of this circuitry produce anterograde memory loss, as has been clearly documented in numerous clinical cases and animal models [22], [39], [42]. Similarly, the subdivisions of amygdala (lateral, basal, central) enriched in PNC in WT animals, and exhibiting reductions in Bbs4^-/-^ mice, play an essential role in the acquisition and expression of conditioned fear responses [43]–[45]. Our analyses clearly demonstrate pronounced pathological alterations of PNC in the amygdala and hippocampus of Bbs4^-/-^ mice. The number of PNC per unit area in these regions was also significantly lower than in corresponding areas of the WT control brain. Our ultrastructural findings revealed that at least a portion of the loss of ACIII immunoreactivity is due to gross structural disorganization and truncation of cilia within these regions. When combined with the consistently lower intensity of staining observed in mutant animals and the statistically significant reduction in levels of ACIII in mutants demonstrated in the Western Blot analysis it is clearly apparent that the signaling capacity of cilia in amygdala and hippocampus of mutant animals is severely compromised. Considered with the aforementioned data demonstrating the essential role of ACIII in the signaling capacity of cilia and the learning deficits associated with deletion of cilia, our data suggest that more detailed behavioral assessment of these processes in Bbs4^-/-^ animals will be informative in clarifying the role of PNC in neuronal signaling underlying these functions. Determining the interrelations of signal transduction through PNC and that achieved through classical synaptic communication is an obvious and important goal for future investigations.

Characterizing the precise pathology that is present in the brain of BBS animal models at early ages [6], and how it may differ in animals with deletions or mutations of the BBS gene family, remains an important goal for defining the pathology present in humans affected with the syndrome. The observation of variable reductions in the number and extent of ACIII immunoreactive cilia among Bbs4^-/-^ mice is consistent with the variable phenotype observed in the human disease; *e.g.*, differences in IQ range (from 42 to 108) in 21 children with BBS [21]. Similarly, the differential reductions of ACIII immunoreactivity in subdivisions of the amygdala and hippocampal formation may interfere with circuit related function of these regions. For example, dysfunction of PNC in the intercalated cell masses may impair the GABA-ergic inputs to the centromedial complex, thereby disrupting influences of the amygdala upon hypothalamic and brainstem centers involved in the elaboration of autonomic responses and species-specific emotional behaviors [46]. The differential alterations in the length of cilia documented in CA1 of dorsal hippocampus in our analysis are intriguing and raise the possibility that alterations of BBS gene expression may have regional consequences for cilia structure and function. In this regard it is interesting to note that increased expression of the dyslexia candidate gene (DCDC2) affects the length and signaling of PNC and hippocampal neuronal morphology [47].

The observed PNC pathology in BBS mutant mice brains may also result because of absence or damage of another important organelle, the ciliary rootlet (CR). It is known that CR is essential for long-term stability of sensory cilia in photoreceptor cells [48] and plays a role in intracellular transport in ciliated cells [49]. It was described in 2002 that the enzyme rootletin is a structural component of the rootlet [50]. Seeley and Nachury noted that the CR is not essential for ciliary biogenesis or function but is integral to imparting mechanical support to cilia [51]. The data published by Yang and colleagues reports that rootletin-null mice display ciliary defects in photoreceptor cells [48]. It is critically important to study the CRs in hippocampus and amygdala of Bbs4^-/-^ brains where the defective cilia were present and compare with the areas where the pathology of PNC have not been observed. A further light microscopic immunocytochemical localizations of rootletin and biochemical analyses of these regions of the brain are necessary to understand if the absence of this enzyme may influence the defective PNC.

**Figure 11:**
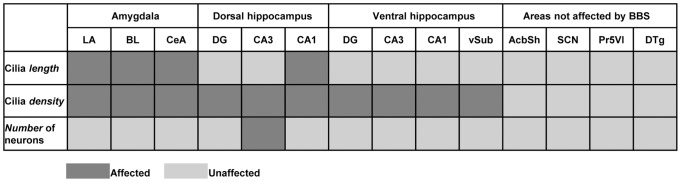
Variable ciliopathy phenotype in the investigated brain regions. Dark grey rectangle corresponds to the regions where ether the PNC or the number of neurons are affected in Bbs4^-/-^ mice brains. Light gray rectangle corresponds to the unaffected areas. Abbreviations as for Figures 1, 4 & 7.

The fact that the reductions in PNC number and ciliopathy observed in the hippocampus and amygdala of Bbs4^-/-^ mice were not generalized to all areas of the brain is an important observation that provides further support for the hypothesis that ciliopathy may contribute to the BBS phenotype (Figure 11). In this regard, the pathology documented in both the human disorder and animal models is concentrated in areas of the brain involved in learning and memory while no pathology has been reported in subcortical regions not directly related to these functions. Our analysis is consistent with these findings in that we demonstrate normal PNC numbers and no overt morphological alterations in PNC in areas of the brain ofBbs4^-/-^ mutants that participate in the regulation of reward and motivation (AcbSh), of circadian rhythms (SCN), of light touch of the face sensation (Pr5VL), of angular head velocity (DTg) and are not known to exhibit abnormal function in the animal model of BBS. Interestingly, all areas of the Bbs4^-/-^ mice brains investigated in this study did not exhibit a significant difference in the number of neurons compared to the same regions of WT mice brains (Figure 11) except for the CA3 of the dorsal hippocampus. Nevertheless, an analysis of the distribution of ACIII+ cilia in the cortical mantle remains an important goal for fully defining the potential role of PNC in the cognitive deficits observed in Bbs null mice. As noted previously, cortical thinning has been documented in BBS and is differentially distributed among cortical regions. Cortical thinning has also been documented in Bbs deletion mutants (e.g., [6], [22]) but has not been subjected to detailed regional characterizations. Whereas the cell loss that associated with the reduction in cortical mass in Bbs deletion mutants certainly contributes to the cognitive deficits exhibited by in these animals it will be important to determine if surviving cortical neurons exhibit a ciliopathy similar to that we have documented in hippocampus and amygdala.

These findings, considered with the differential concentration of ciliopathy in areas of Bbs4^-/-^ brain that mediate cognition and learning (the other areas to be investigated), provide a strong rationale for a more detailed functional analysis of the role of cilia in neuronal communication as well as the role that ciliopathy may play in the genesis of the symptoms characteristic of BBS.

## Experimental Procedures

### 2.1. Animals

Ten adult Bbs4^-/-^ male and eight littermate control (WT) male mice were included in the study. The animals were produced and housed at the University of Iowa as described previously [19]. The genotype of each animal was determined with PCR according to previously published procedures [16], [17]. Animals were anesthetized and perfused at the University of Iowa and the Immunohistochemical, light and electron microscopic processing of the brains was conducted at the University of Pittsburgh. The University of Iowa Animal Research Committee approved all procedures and regulations relevant to the collection and processing of tissue. Additionally, all experimental procedures conformed to the NIH's Guidelines for the Care and Use of Laboratory Animals.

### 2.2. Anesthesia and perfusion

Eight Bbs4^-/-^ and six WT mice were anesthetized with an intraperitoneal injection of ketamine (91 mg/kg) and xylazine (9.1 mg/kg), and all efforts were made to minimize suffering. Deeply anesthetized animals were then perfused transcardially with phosphate buffered saline (PBS) followed by 4% paraformaldehyde and 0.5% glutaraldehyde in PBS for light and by 2% paraformaldehyde and 1.0% glutaraldehyde in PBS for electron microscopic studies. The brains were removed and postfixed in the same fixative overnight at 4°C. Fixed brains were rinsed in PBS and transferred to a solution containing sucrose, Polyvinil-pyrrolidone, ethylene glycol and 0.1 M sodium phosphate buffer [52] for cryotprotection and stored at −20°C.

### 2.3. Immunohistochemistry

#### 2.3.1. ACIII antibody specificity

The rabbit polyclonal antibody used for ACIII localization was purchased from Santa Cruz Biotechnology (Santa Cruz, CA; catalog number sc-588). It was prepared against a synthetic peptide identical to the C terminal 20 amino acids of mouse ACIII. The antiserum recognizes a band of approximately 200 kD on Western blot and does not produce an immunocytochemical signal in tissue from ACIII knockout mice (Wong et al., 2000). Bishop and colleagues (2007) demonstrated that immunocytochemical staining of tissue from 129:BL6 mice was eliminated by preabsorbtion of the antiserum with the immunizing peptide (Santa Cruz; catalog number sc-588P). We confirmed this observation using sections from WT and Bbs4^-/-^ brains. Preabsorbtion of the primary antiserum with the blocking peptide completely abolished immunostaining in WT and Bbs4^-/-^ brain sections.

#### 2.3.2. Immunohistochemical Processing

Cryoprotected brains were sectioned in the coronal plane (35 μm) using a Leica SM200R sliding microtome equipped with a tissue freezing stage. The coronal sections were collected in PBS containing 0.3% Triton-X (PBT) and then processed for immunohistochemical localization of ACIII using the glucose oxidase-DAB-nickel method [53]. Brain sections from the control and transgenic mice were processed and rinsed using the same solutions and the incubation times were equivalent such that all the sections were developed simultaneously in the same conditions. Tissue was incubated in a 1∶500 dilution of the primary antibody for 48 hours at 4°C. Following washes in PBT for 1 hour the sections were incubated in a 1∶200 dilution of donkey anti-rabbit secondary antibody (Jackson ImmunoResearch Laboratories, Inc.; catalog number 711-065-152) diluted in PBT. After a 30-minute rinse in multiple changes of PBT the sections were incubated in Vectastain *Elite* reagents (Vector Laboratories, Burlingame, CA) for 2 hours. Sections were rinsed in 0.1 molar acetate buffer (pH 6.0) and the reaction product was generated by incubating the sections in a glucose oxidase-DAB-nickel solution. Thereafter, the tissue was rinsed with 0.1M acetate buffer, transferred to PBS, mounted on Superfrost Plus microscope slides (Fisher Scientific, Pittsburgh, PA) and, in some cases, counterstained with neutral red that visualizes neuronal cell bodies ([NDT206-FD]) and was used to label neurons in the rodent brain [54]. Sections were dehydrated in graded ethanols, cleared in xylenes, and coverslipped with Cytoseal 60 (Richard-Allan Scientific, Kalamazoo, MI). All sections were photographed using bright field optics and an Olympus BX51 microscope equipped with a Hamamatsu ORCA-ER digital camera.

### 2.4. Electron microscopy

Vibratome 50 μm sections were postfixed in 1% osmium tetroxide, washed in repeated changes of 0.1 M PB, and dehydrated in a graded ethanol series. The sections were then passed through multiple changes of acetone followed by sequential changes of increasing concentrations of Epon-Araldite embedding media diluted in acetone (1∶1 for 6 h; 3∶1 overnight). After a final change in 100% resin for 12 h the sections were flat-embedded and polymerized overnight at 60°C. The areas of interest were dissected from the flat-embedded sections and thin sections were cut using a Leika Ultracut R ultramicrotome. Ultrathin sections were examined with a Morgagni transmission electron microscope and the images were taken by a digital camera attached to the microscope.

### 2.5. Western blot analysis of ACIII

Two Bbs4^-/-^ and two WT mice were used for Western blot analysis. The animals were anesthetized as described in section 4.2 and decapitated. The brains were quickly removed and placed in liquid nitrogen. The areas of interest were later dissected and placed in numbered tubules. Western blots of hippocampus, amygdala, nucleus accumbens shell and suprachiasmatic nuclei from wild type and Bbs4^-/-^ mice using the same antibody utilized to immunohistochemically detect ACIII peptide. The Western blots were performed by an investigator who was blinded to sample identity. Densitometric analyses of the immunoblots were performed using the ImageJ software.

### 2.6. Data Analysis

Qualitative evaluations of the relative number and morphology of ACIII immunopositive cilia throughout the hippocampus, amygdala, AcbSh, SCN, DTg and Pr5VL were made on every section that contained these brain subdivisions. Quantitative analysis was conducted using single sections that passed through the intermediate portion of the central nucleus of the amygdala (Figures 1 B & C), through the rostral portion of hippocampus that contains dorsal hippocampal subfields (Figures 3 B & C), through the caudal portion of the hippocampus that contains both dorsal and ventral hippocampal subfields (Figures 4 A–C), through the middle part of AcbSh and through sections containing the SCN, DTg and Pr5VL (Figure 6). Matching sections were selected for each case and corresponded to the following coordinates of the Mouse Brain in Stereotaxic Coordinates atlas [55]: 0.98–1.18 rostral to Bregma for AcbSh; 0.46–0.58 caudal to Bregma for SCN; 1.22–1.58 caudal to Bregma for amygdala, 1.70–1.94 caudal to Bregma for dorsal hippocampus, 3.28–3.52 caudal to Bregma for ventral hippocampus; 5.02 caudal to Bregma for DTg and Pr5VL.

Cilia were counted within these areas at all focal points using a 40× objective (Nikon Optiphot microscope equipped with a MTI DC330E digital camera) and StereoInvestigator image analysis software (Version 10; MicroBrightField, Williston, VT). Subdivisions of each region of amygdala, hippocampus, AcbSh, SCN, DTg and Pr5VL were subsequently defined and the counts of immunopositive cilia within each subfield were determined. To normalize for variations in the size of the regions between knockout and wild type animals, the number of immunopositive cilia in each subdivision was divided by the area and reported as the density of cilia within each region (cilia/μm^2^). We decided not to divide the SCN, because no difference was detected between the dorsomedial and ventrolateral subdivisions. Cilia length was computed from 40× images of subdivisions of dorsal and ventral hippocampus using Image J software (http://rsbweb.nih.gov/ij/).

Numbers of neurons within each of the regions subjected to analysis of immunopositive cilia were also computed. Counts were made using the optical dissector probe of Stereo Investigator software. Each region was outlined and neurons exhibiting a stained nucleolus were marked in the randomly sampled fields selected by the software. Guard zones within each sampled area were excluded from counting. To normalize for variations in the size of the regions between knockout and wild type animals, the number of neurons in each subdivision was divided by the area and reported as the density of neurons within each region (neurons/μm^2^).

### 2.7. Statistical analysis

Data were analyzed using a repeated measures ANOVA tests, with brain region as the repeated (within subjects) measure, and genotype as the between subjects factor. Demonstration of significant effects in the ANOVA analysis was followed with post hoc Student's *t*-tests to determine if statistically significant differences in cilia density or length of cilia existed between control and mutant mice within comparable regions of the brain. Differences between regions were considered significant when the P value was ≤0.05.

## References

[1] Laurence JZ, Moon RC (1866) Four cases of “retinitis pigmentosa” occurring in the same family and accompanied by general imperfection of development. Ophtal Rev 2: 32–41. 10.1002/j.1550-8528.1995.tb00166.x 8521157

[2] Bardet G (1920) Sur un Syndrome d'Obésité infantile avec Polydactylie et Rétinite pigmentaire (Contribution à l'Étude des Formes cliniques de l'Obésité hypophysaire). Thesis No.479.

[3] Biedl A (1922) Ein Geschwisterpaar mit adiposo-genitaler Dystrofie. Deutsch Med Wochenschr 48: 1630.

[4] Fliegauf M, Benzing T, Omran H (2007) When cilia go bad: cilia defects and ciliopathies. Nat Rev Mol Cell Biol 8: 880–893. 17955020 10.1038/nrm2278

[5] Keppler-Noreuil K, Blumhorst C, Sapp J, Brinckman D, Johnston J, et al (2011) Brain tissue- and region-specific abnormalities on volumetric MRI scans in 21 patients with Bardet-Biedl syndrome (BBS). BMC Medical Genetics 12: 101. 21794117 10.1186/1471-2350-12-101 PMC3199749

[6] Swiderski RE, Agassandian K, Ross JL, Bugge K, Cassell MD, et al (2012) Structural defects in cilia of the choroid plexus, subfornical organ and ventricular ependyma are associated with ventriculomegaly. Fluids Barriers CNS 9: 22–35 2045-8118-9-22. 23046663 10.1186/2045-8118-9-22 PMC3527152

[7] Sheffield VC, Zhang Q, Heon E, Stone EM, Carmi R (2008) The Bardet-Biedl Syndromes.

[8] Loktev AV, Zhang Q, Beck JS, Searby CC, Scheetz TE, et al (2008) A BBSome Subunit Links Ciliogenesis, Microtubule Stability, and Acetylation. Developmental Cell 15: 854–865. 19081074 10.1016/j.devcel.2008.11.001

[9] Nachury MV, Loktev AV, Zhang Q, Westlake CJ, Peränen J, et al (2007) A Core Complex of BBS Proteins Cooperates with the GTPase Rab8 to Promote Ciliary Membrane Biogenesis. Cell 129: 1201–1213. 17574030 10.1016/j.cell.2007.03.053

[10] Scheidecker S, Etard C, Pierce NW, Geoffroy V, Schaefer E, et al.. (2013) Exome sequencing of Bardet-Biedl syndrome patient identifies a null mutation in the BBSome subunit BBIP1 (BBS18). Journal of Medical Genetics (online). 10.1136/jmedgenet-2013-101785 PMC3966300 24026985

[11] Jin H, White SR, Shida T, Schulz S, Aguiar M, et al (2010) The Conserved Bardet-Biedl Syndrome Proteins Assemble a Coat that Traffics Membrane Proteins to Cilia. Cell 141: 1208–1219. 20603001 10.1016/j.cell.2010.05.015 PMC2898735

[12] Domire J, Green J, Lee K, Johnson A, Askwith C, et al.. (2010) Dopamine receptor 1 localizes to neuronal cilia in a dynamic process that requires the Bardet-Biedl syndrome proteins. Cellular and Molecular Life Sciences 1–10. 10.1007/s00018-010-0603-4 PMC3368249 21152952

[13] Berbari NF, Lewis JS, Bishop GA, Askwith CC, Mykytyn K (2008) Bardet-Biedl syndrome proteins are required for the localization of G protein-coupled receptors to primary cilia. Proc Natl Acad Sci USA 105: 4242–4246. 18334641 10.1073/pnas.0711027105 PMC2393805

[14] Seo S, Baye LM, Schulz NP, Beck JS, Zhang Q, et al (2010) BBS6, BBS10, and BBS12 form a complex with CCT/TRiC family chaperonins and mediate BBSome assembly. Proc Natl Acad Sci U S A 107: 1488–1493. 20080638 10.1073/pnas.0910268107 PMC2824390

[15] Fath MA, Mullins RF, Searby C, Nishimura DY, Wei J, et al (2005) Mkks-null mice have a phenotype resembling Bardet-Biedl syndrome. Hum Mol Genet 14: 1109–1118. 15772095 10.1093/hmg/ddi123

[16] Mykytyn K, Mullins RF, Andrews M, Chiang AP, Swiderski RE, et al (2004) Bardet-Biedl syndrome type 4 (BBS4)-null mice implicate Bbs4 in flagella formation but not global cilia assembly. Proceedings of the National Academy of Sciences of the United States of America 101: 8664–8669. 15173597 10.1073/pnas.0402354101 PMC423252

[17] Nishimura DY, Fath M, Mullins RF, Searby C, Andrews M, et al (2004) Bbs2-null mice have neurosensory deficits, a defect in social dominance, and retinopathy associated with mislocalization of rhodopsin. Proc Natl Acad Sci U S A 101: 16588–16593. 15539463 10.1073/pnas.0405496101 PMC534519

[18] Ross AJ, May-Simera H, Eichers ER, Kai M, Hill J, et al (2005) Disruption of Bardet-Biedl syndrome ciliary proteins perturbs planar cell polarity in vertebrates. Nat Genet 37: 1135–1140. 16170314 10.1038/ng1644

[19] Rahmouni K, Fath MA, Seo S, Thedens DR, Berry CJ, et al (2008) Leptin resistance contributes to obesity and hypertension in mouse models of Bardet-Biedl syndrome. Journal of Clinical Investigation 118: 1458–1467. 18317593 10.1172/JCI32357 PMC2262028

[20] Eichers ER, Abd-El-Barr MM, Paylor R, Lewis RA, Bi W, et al (2006) Phenotypic characterization of Bbs4 null mice reveals age-dependent penetrance and variable expressivity. Hum Genet 120: 211–226. 16794820 10.1007/s00439-006-0197-y

[21] Barnett S, Reilly S, Carr L, Ojo I, Beales PL, et al (2002) Behavioural phenotype of Bardet-Biedl syndrome. J Med Genet 39: e76. 12471214 10.1136/jmg.39.12.e76 PMC1757216

[22] Baker K, Northam GB, Chong WK, Banks T, Beales P, et al (2011) Neocortical and hippocampal volume loss in a human ciliopathy: A quantitative MRI study in Bardet-Biedl syndrome. Am J Med Genet 155: 1–8. 10.1002/ajmg.a.33773 21204204

[23] Davis RE, Swiderski RE, Rahmouni K, Nishimura DY, Mullins RF, et al (2007) A knockin mouse model of the Bardet-Biedl syndrome 1 M390R mutation has cilia defects, ventriculomegaly, retinopathy, and obesity. Proc Natl Acad Sci U S A 104: 19422–19427. 18032602 10.1073/pnas.0708571104 PMC2148305

[24] Carter CS, Vogel TW, Zhang Q, Seo S, Swiderski RE, et al (2012) Abnormal development of NG2+PDGFR-alpha+ neural progenitor cells leads to neonatal hydrocephalus in a ciliopathy mouse model. Nat Med 18: 1797–1804. 23160237 10.1038/nm.2996 PMC3684048

[25] Chamling X, Seo S, Bugge K, Searby C, Guo DF, et al (2013) Ectopic Expression of Human BBS4 Can Rescue Bardet-Biedl Syndrome Phenotypes in Bbs4 Null Mice. PLoS ONE 8: e59101. 23554981 10.1371/journal.pone.0059101 PMC3598656

[26] Bishop GA, Berbari NF, Lewis J, Mykytyn K (2007) Type III adenylyl cyclase localizes to primary cilia throughout the adult mouse brain. J Comp Neurol 505: 562–571. 17924533 10.1002/cne.21510

[27] Praetorius HA, Spring KR (2005) A physiological view of the primary cilium. Annual Review of Physiology 67: 515–529. 10.1146/annurev.physiol.67.040403.101353 15709968

[28] Amador-Arjona A, Elliott J, Miller A, Ginbey A, Pazour GJ, et al (2011) Primary Cilia Regulate Proliferation of Amplifying Progenitors in Adult Hippocampus: Implications for Learning and Memory. The Journal of Neuroscience 31: 9933–9944. 21734285 10.1523/JNEUROSCI.1062-11.2011 PMC3758574

[29] Badano JL, Mitsuma N, Beales PL, Katsanis N (2006) The Ciliopathies: An Emerging Class of Human Genetic Disorders. Annu Rev Genom Human Genet 7: 125–148. 10.1146/annurev.genom.7.080505.115610 16722803

[30] Baker K, Beales PL (2009) Making sense of cilia in disease: the human ciliopathies. Am J Med Genet C Semin Med Genet 151C: 281–295. 19876933 10.1002/ajmg.c.30231

[31] Beales PL, Elcioglu N, Woolf AS, Parker D, Flinter FA (1999) New criteria for improved diagnosis of Bardet-Biedl syndrome: results of a population survey. J Med Genet 36: 437–446. 10874630 PMC1734378

[32] Rooryck C, Pelras S, Chateil JF, Cances C, Arveiler B, et al (2007) Bardet-biedl syndrome and brain abnormalities. Neuropediatrics 38: 5–9. 17607597 10.1055/s-2007-981466

[33] Leroux MR (2007) Taking vesicular transport to the cilium. Cell 129: 1041–1043. 17574016 10.1016/j.cell.2007.05.049

[34] Veland IR, Awan A, Pedersen LB, Yoder BK, Christensen ST (2009) Primary cilia and signaling pathways in mammalian development, health and disease. Nephron Physiol 111: 39–53. 10.1159/000208212 PMC2881330 19276629

[35] Keryer G, Pineda JR, Liot G, Kim J, Dietrich P, et al (2011) Ciliogenesis is regulated by a huntingtin-HAP1-PCM1 pathway and is altered in Huntington disease. J Clin Invest 121: 4372–4382. 21985783 10.1172/JCI57552 PMC3223861

[36] Alberts B, Johnson A, Lewis J, Raff M, Bray D, et al. (2004) *Essential cell biology* (2 ed.). New York: Garland Science.

[37] Rosenbaum JL, Witman GB (2002) Intraflagellar transport. Nat Rev Mol Cell Biol 3: 813–825. 12415299 10.1038/nrm952

[38] Wong ST, Trinh K, Hacker B, Chan GCK, Lowe G, et al (2000) Disruption of the Type III Adenylyl Cyclase Gene Leads to Peripheral and Behavioral Anosmia in Transgenic Mice. Neuron 27: 487–497. 11055432 10.1016/s0896-6273(00)00060-x

[39] Wang Z, Phan T, Storm DR (2011) The Type 3 Adenylyl Cyclase Is Required for Novel Object Learning and Extinction of Contextual Memory: Role of cAMP Signaling in Primary Cilia. The Journal of Neuroscience 31: 5557–5561. 21490195 10.1523/JNEUROSCI.6561-10.2011 PMC3091825

[40] Guadiana SM, Semple-Rowland S, Daroszewski D, Madorsky I, Breunig JJ, et al (2013) Arborization of Dendrites by Developing Neocortical Neurons Is Dependent on Primary Cilia and Type 3 Adenylyl Cyclase. The Journal of Neuroscience 33: 2626–2638. 23392690 10.1523/JNEUROSCI.2906-12.2013 PMC6619186

[41] Kumamoto N, Gu Y, Wang J, Janoschka S, Takemaru K, et al (2012) A role for primary cilia in glutamatergic synaptic integration of adult-born neurons. Nat Neurosci 15: 399–405. 22306608 10.1038/nn.3042 PMC3288565

[42] Zola-Morgan S, Squire LR, Amaral DG (1986) Human amnesia and the medial temporal region: enduring memory impairment following a bilateral lesion limited to field CA1 of the hippocampus. J Neurosci 6: 2950–2967. 3760943 10.1523/JNEUROSCI.06-10-02950.1986 PMC6568782

[43] Cardinal RN, Parkinson JA, Hall J, Everitt BJ (2002) Emotion and motivation: the role of the amygdala, ventral striatum, and prefrontal cortex. Neuroscience & Biobehavioral Reviews 26: 321–352. 12034134 10.1016/s0149-7634(02)00007-6

[44] Ledoux JE (2000) Emotion Circuits in the Brain. Annual Review of Neuroscience 23: 155–184. 10.1146/annurev.neuro.23.1.155 10845062

[45] Sah P, Faber ES, Lopez DA, Power J (2003) The amygdaloid complex: anatomy and physiology. Physiol Rev 83: 803–834. 12843409 10.1152/physrev.00002.2003

[46] Paré D, Smith Y (1993) The Intercalated Cell Masses Project to the Central and Medial Nuclei of the Amygdala in Cats. Neuroscience 57: 1077–1090. 8309544 10.1016/0306-4522(93)90050-p

[47] Massinen S, Hokkanen ME, Matsson H, Tammimies K, Tapia-Paez I, et al (2011) Increased Expression of the Dyslexia Candidate Gene DCDC2 Affects Length and Signaling of Primary Cilia in Neurons. PLoS ONE 6: e20580. 21698230 10.1371/journal.pone.0020580 PMC3116825

[48] Yang J, Gao J, Adamian M, Wen XH, Pawlyk B, et al (2005) The Ciliary Rootlet Maintains Long-Term Stability of Sensory Cilia. Molecular and Cellular Biology 25: 4129–4137. 15870283 10.1128/MCB.25.10.4129-4137.2005 PMC1087714

[49] Yang J, Li T (2005) The ciliary rootlet interacts with kinesin light chains and may provide a scaffold for kinesin-1 vesicular cargos. Experimental Cell Research 309: 379–389. 16018997 10.1016/j.yexcr.2005.05.026

[50] Yang J, Liu X, Yue G, Adamian M, Bulgakov O, et al (2002) Rootletin, a novel coiled-coil protein, is a structural component of the ciliary rootlet. J Cell Biol 159: 431–440. 12427867 10.1083/jcb.200207153 PMC2173070

[51] Seeley ES, Nachury MV (2010) The perennial organelle: assembly and disassembly of the primary cilium. Journal of Cell Science 123: 511–518. 20144999 10.1242/jcs.061093 PMC2818191

[52] Watson RE Jr, Wiegand SJ, Clough RW, Hoffman GE (1986) Use of cryoprotectant to maintain long-term peptide immunoreactivity and tissue morphology. Peptides 7: 155–159. 10.1016/0196-9781(86)90076-8 3520509

[53] Shu SY, Ju G, Fan LZ (1988) The glucose oxidase-DAB-nickel method in peroxidase histochemistry of the nervous system. Neurosci Lett 85: 169–171. 3374833 10.1016/0304-3940(88)90346-1

[54] Peyron C (1998) Neurons containing hypocretin (orexin) project to multiple neuronal systems. The Journal of Neuroscience 18: 9996–10015. 9822755 10.1523/JNEUROSCI.18-23-09996.1998 PMC6793310

[55] Franklin KBJ, Paxinos G (1997) The mouse brain in stereotaxic coordinates. 216 pp.

